# Facile synthesis of FeCeO_x_ nanoparticles encapsulated carbon nitride catalyst for highly efficient and recyclable synthesis of substituted imidazoles

**DOI:** 10.1038/s41598-023-44747-7

**Published:** 2023-10-14

**Authors:** Najmedin Azizi, Mostafa Saadat, Mahtab Edrisi

**Affiliations:** Chemistry and Chemical Engineering Research Center of Iran, P.O. Box 14335-186, Tehran, Iran

**Keywords:** Catalysis, Green chemistry

## Abstract

Herein, we developed a novel composite called FeCeO_x_@g-C_3_N_4_ through a combination of sonication, sintering, and hydrothermal techniques to implement the principles of green chemistry by utilizing reusable nanocomposites in one-pot reactions. To gain a comprehensive understanding of the catalyst's structure, composition, and morphology, various characterization methods were employed. These included FT-IR analysis to examine chemical bonds, SEM and TEM imaging to visualize the catalyst's surface and internal structure, TGA to assess thermal stability, EDS for elemental composition analysis, and XRD to determine crystal structure. The FeCeO_x_@g-C_3_N_4_ nanocatalyst demonstrated remarkable efficacy in the one-pot synthesis of 2,4,5-trisubstituted and 1,2,4,5-tetrasubstituted imidazole. Noteworthy features of this catalyst included high percentage yield, mild reaction conditions, short reaction time, and an efficient and straightforward procedure. Furthermore, the FeCeO_x_@g-C_3_N_4_ composite exhibited excellent recyclability and reusability. It could be recycled and reused up to four times without a significant decline in catalytic activity.

## Introduction

Imidazole derivatives have gained significant importance due to their biological activity in natural products, biology, intermediates, and pharmacologically active compounds^1^. The presence of a lone pair of nitrogen in the imidazole ring enables the formation of hydrogen bonding, which contributes to their metal-binding capability. This property has found applications in the pharmaceutical industry^2–5^. In recent years, imidazole compounds have garnered widespread attention due to their diverse range of properties and applications. These compounds exhibit antibiotic, anti-tumoral, pesticide, herbicide, anti-allergy, anti-viral, and other pharmacological activities^6,7^. Moreover, the imidazole structure is present in various drugs such as losartan, eprosartan, histidine, and histamine^8^.

Two important imidazole derivatives are 1,2,4,5-tetraphenylimidazole and 2,4,5-triphenylimidazole. The first imidazole compound was reported by Radzisewski, Japp, and Robinson in 1882. They achieved its synthesis by reacting 1,2-dialdehyde with ammonium chloride. Since then, several imidazoles have been reported, derived from 1,2-diketones, α-ketomonoximes, α-hydroxy ketones in combination with aldehydes and ammonium^9,10^. These imidazole derivatives have found numerous applications and gained importance across various industries^11^. Considering the significant applications of imidazole derivatives, several synthetic protocols with high yield and efficiency have been documented in the literature^12^. However, some of these protocols utilize toxic and expensive catalysts that are less efficient. Consequently, they often involve harsh reaction conditions, long reaction times, and yield limitations^13–15^.

Graphitic carbon nitride (g-C_3_N_4_) has garnered significant attention and found applications in various energy-related fields^16^. g-C_3_N_4_ primarily consists of carbon and nitrogen atoms and possesses desirable properties such as easy synthesis and functionalization, excellent physicochemical stability, wide bandgap, low toxicity, and cost-effectiveness. The unique properties of g-C_3_N_4_, such as its functionalization capabilities, physicochemical stability, and low cost, make it an attractive choice for supporting other catalysts or functional materials^17–21^. One key advantage of g-C_3_N_4_ is its covalent bond nature, which results in an inactive surface that reduces the interaction between hydrogen and oxygen^22–24^. This characteristic enhances its catalytic properties^19,25,26^. However, pure g-C_3_N_4_ has two main limitations: first, it absorbs only a small fraction of solar energy, primarily in the range of low bandgap wavelengths (below 460 nm). Second, the fast recombination of double electrons within the cavities of g-C_3_N_4_ leads to a decrease in photocatalytic activity^27–30^. To address these limitations, various approaches have been developed to enhance the catalytic activity of g-C_3_N_4_ and mitigate imperfections^31^. These approaches include doping g-C_3_N_4_ with transition metals^32–35^ and coupling it with metals^36–41^. These strategies aim to improve the absorption of a broader range of solar energy, reduce electron recombination, and enable the recovery and recycling of g-C_3_N_4_, thereby enhancing its overall performance as a catalyst^42–44^.

Magnetic materials offer a range of advantages due to their inherent magnetism and unique properties^45,46^. They are crucial in various industries and applications^47^. Magnetic storage devices rely on their ability to retain magnetization, enabling high-capacity data storage^48^. Electric motors and generators utilize magnetic materials for efficient energy conversion^49^. Magnetic sensors enable precise detection and measurement of magnetic fields in compasses, position sensing, and current sensing^50^. In industries like mining and recycling, magnetic materials facilitate effective separation techniques^51^. Biomedical applications benefit from magnetic nanoparticles in imaging, drug delivery, and cancer treatment^52^. Magnetic materials also play a vital role in non-destructive testing and offer versatility for customization^53^.

In our continue interest to the expanding knowledge of carbon nitride-based catalysts and their application in various organic transformations^54–56^, herein, we successfully prepared FeCeO_x_@g-C_3_N_4_ nanocomposites, which serve as a novel catalyst with excellent activity in the one-pot synthesis of 1,2,4,5-tetraphenylimidazole and 2,4,5-triphenylimidazole derivatives under mild reaction conditions. By combining FeCeO_x_ with g-C_3_N_4_, we have fabricated a catalyst with enhanced performance and activity that offer practical benefits, such as energy efficiency and environmental friendliness.

## Results and discussion

### Catalyst characterization

#### SEM

**Figure 1:**
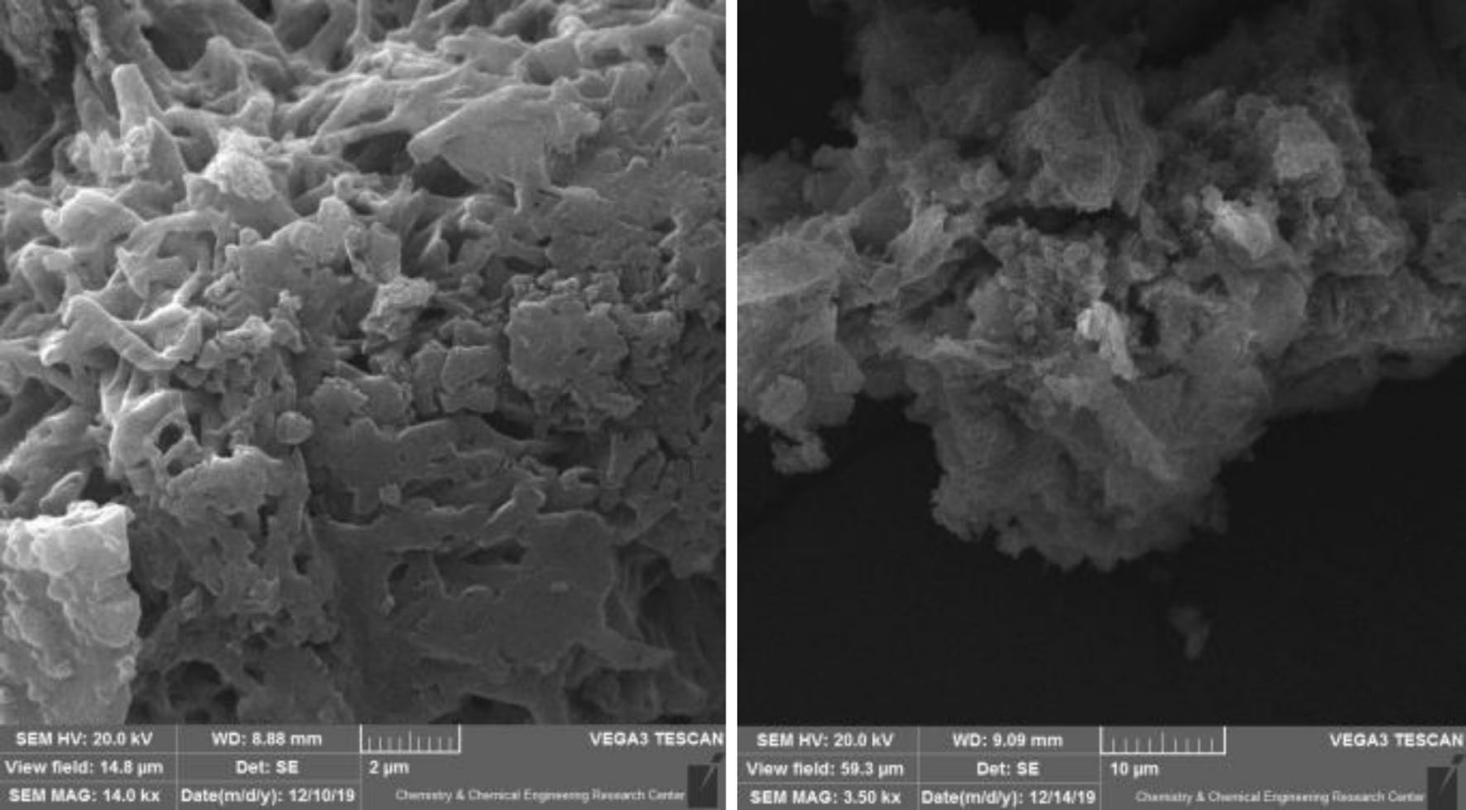
SEM images of FeCeO_x_@g-C_3_N_4_ nanocomposite.

The morphology and microstructure of the FeCeO_x_@g-C_3_N_4_ nanocomposite were investigated using SEM analysis. The obtained SEM images of FeCeO_x_@g-C_3_N_4_ clearly demonstrate the deposition of FeCeO_x_ on the surface of g-C_3_N_4_, as shown in Fig. 1. The SEM images reveal a 2D sheet-like network structure with a uniform distribution of Fe and Ce species on the surface of g-C_3_N_4_. This indicates successful incorporation of FeCeO_x_ onto the g-C_3_N_4_ framework. Importantly, the absence of aggregated species suggests good dispersion and adherence of FeCeO_x_ nanoparticles on the g-C_3_N_4_ surface.

#### EDS

**Figure 2:**
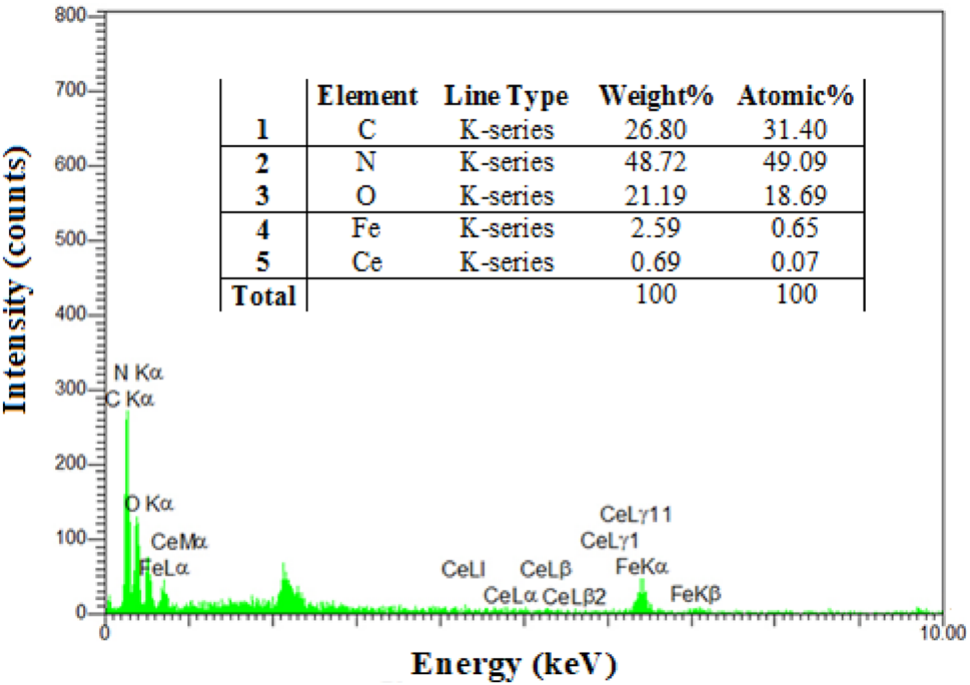
The EDS analysis of FeCeO_x_@g-C_3_N_4_ nanocomposite.

The chemical composition of FeCeO_x_@g-C_3_N_4_ was further confirmed through EDS spectrum analysis. The EDS spectrum, as shown in Fig. 2, reveals the presence of elements associated with Ce, Fe, O, N, and C. The appearance of these elements in the EDS spectrum provides strong evidence for the incorporation of Ce and Fe in the nanocomposite. The presence of Ce indicates the successful integration of CeO_x_, while Fe confirms the presence of FeO_x_. This supports the earlier observations from SEM analysis, indicating the successful deposition of FeCeO_x_ nanoparticles onto the g-C_3_N_4_ framework.

#### XRD

**Figure 3:**
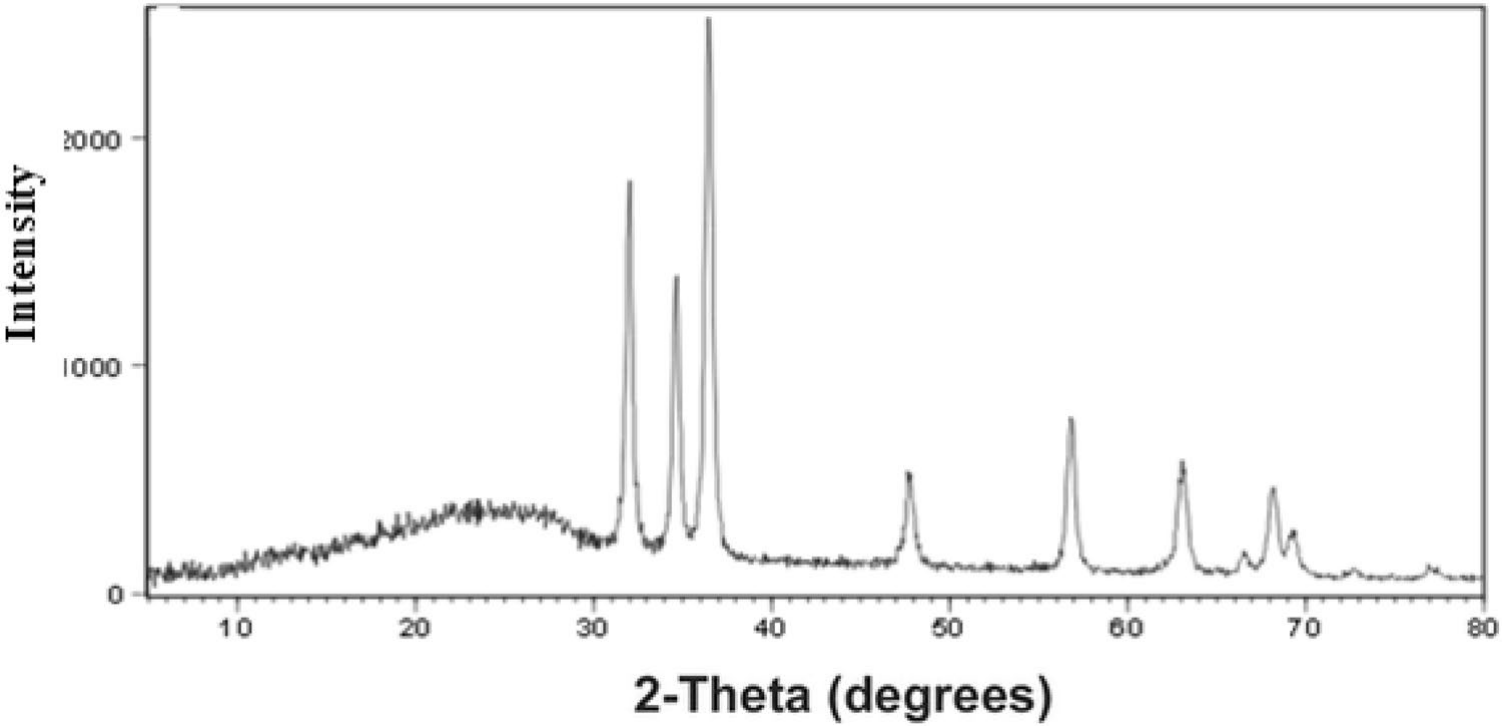
XRD spectra of FeCeO_x_@g-C_3_N_4_ nanocomposite.

The crystal structure of the composite was characterized using a powder X-ray diffractometer (XRD). As shown in Fig. 3, five reflections are observed in the XRD pattern of Fe_2_O_3_ 26.9°, 35.42°, 43.3°, 56.1° and 61.3° that belong to the (2 2 0), (3 1 1), (4 0 0), (5 1 1) and (4 4 0) plane diffractions of Fe_2_O_3_. In addition to the observed reflection at (3 1 1), (2 2 0), (2 0 0), and (1 1 1) belongs to CeO_2_. According to the typical characteristic diffraction peaks of g-C_3_N_4_, two characteristic diffraction peaks can be found at 13.1° and 27.4°.

#### FT-IR

**Figure 4:**
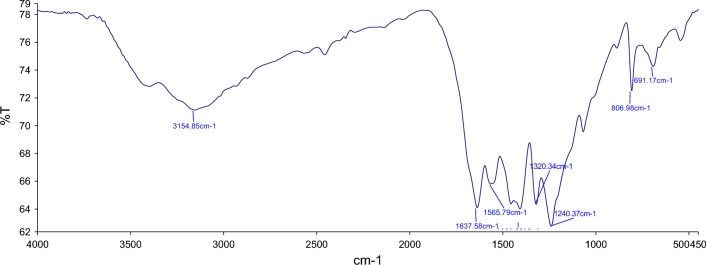
The FT-IR spectra of FeCeO_x_@g-C_3_N_4_ nanocomposite.

FT-IR spectroscopy was employed to investigate the bonding states of the FeCeO_x_@g-C_3_N_4_ nanocomposite. The FT-IR analysis confirms the presence of FeCeO_x_ on g-C_3_N_4_. In Fig. 4, several absorption bands are observed, each corresponding to specific bonding vibrations. The strong absorption band at 580 cm^−1^ is attributed to the Fe–O stretching mode, providing evidence for the presence of FeO in the nanocomposite. Additionally, the absorption bands at 740 cm^−1^ and 1416 cm^−1^ are assigned to the Ce–O stretching vibrations, further confirming the presence of CeO_2_. The absorption band at 804 cm^−1^ corresponds to the bending vibration of the s-triazine ring in g-C_3_N_4_, indicating the presence of g-C_3_N_4_ in the nanocomposite. The absorption bands in the range of 1200–1400 cm^−1^ are attributed to the C–N stretching vibration mode present in g-C_3_N_4_. Furthermore, the absorption band at 1637 cm^−1^ corresponds to the C=N stretching vibration mode, providing further evidence of the presence of g-C_3_N_4_ in the nanocomposite. The broad absorption band observed in the range of 2800–3500 cm^−1^ is indicative of the N–H stretching vibrations of amine groups in g-C_3_N_4_ and the O–H stretching vibrations of absorbed water from the environment.

**Figure 5:**
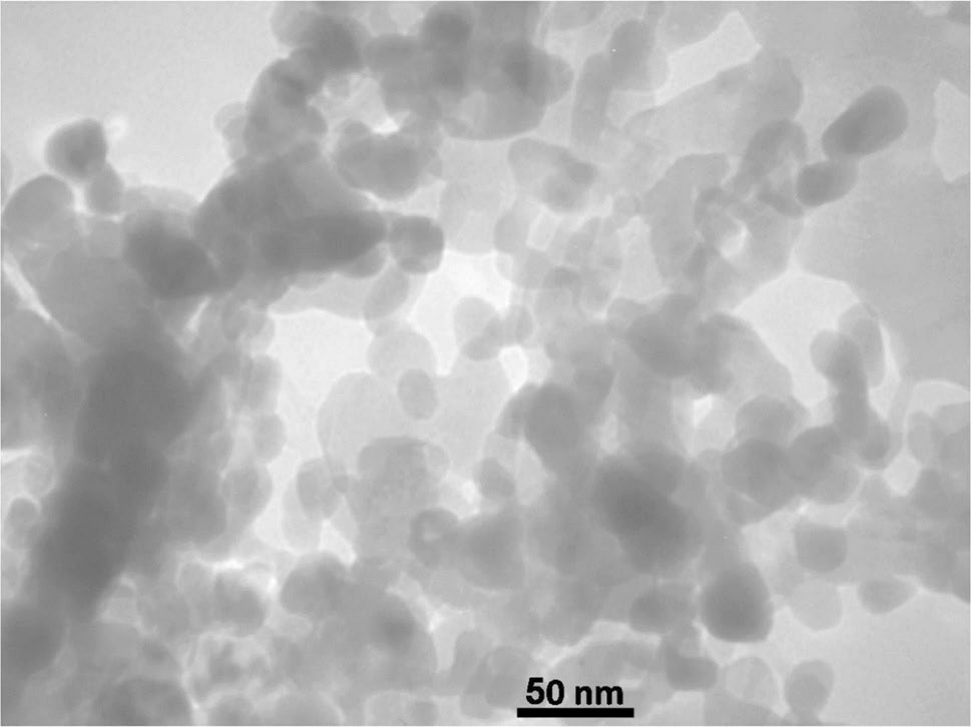
The TEM image of FeCeO_x_@g-C_3_N_4_ nanocomposite.

The detailed morphology and structure of the nanocomposite at the nanoscale level were showed in the TEM image of the FeCeO_x_@g-C_3_N_4_ nanocomposite (Fig. 5). The image reveal FeCeOx particles had good dispersity and uniform distribution of particle size on the surface of the g-C_3_N_4_ matrix. The g-C_3_N_4_ matrix, on the other hand, would appear as a continuous network of interconnected sheets or layers, forming a 2D structure. The g-C_3_N_4_ layers might exhibit a darker contrast compared to the nanoparticles, providing a contrasting background in the TEM image.

Measurement of the surface area of cavities in porous materials is important. Therefore, for the synthesized FeCeO_x_@g-C_3_N_4_ nanocomposite, the surface area, pore volumes and pore size was measured and the obtained data are depicted in Table 1 and supporting information. Specific surface area of pure g-C_3_N_4_ is 41.14 m^2^ g^−1^. The specific surface area of the FeCeO_x_/g-C_3_N_4_ sample, calculated using the BET equation, is 36.12 m^2^ g^−1^, which is lower compared to that of bare g-C_3_N_4_. The decrease of specific surface area along with lower pore volume indicates FeCeOx is loaded to g-C_3_N_4_.

**Table 1:** BET analysis summary of pure g-C_3_N_4_ and FeCeO_x_@g-C_3_N_4_.

Sample	Surface area (m^2^ g^−1^)	Pore vol. (cm^3^ g^−1^)	Pore radius (nm)
g-C_3_N_4_	41.14	0.189	18.4
FeCeO_x_@g-C_3_N_4_	36.12	0.147	12.9

**Figure 6:**
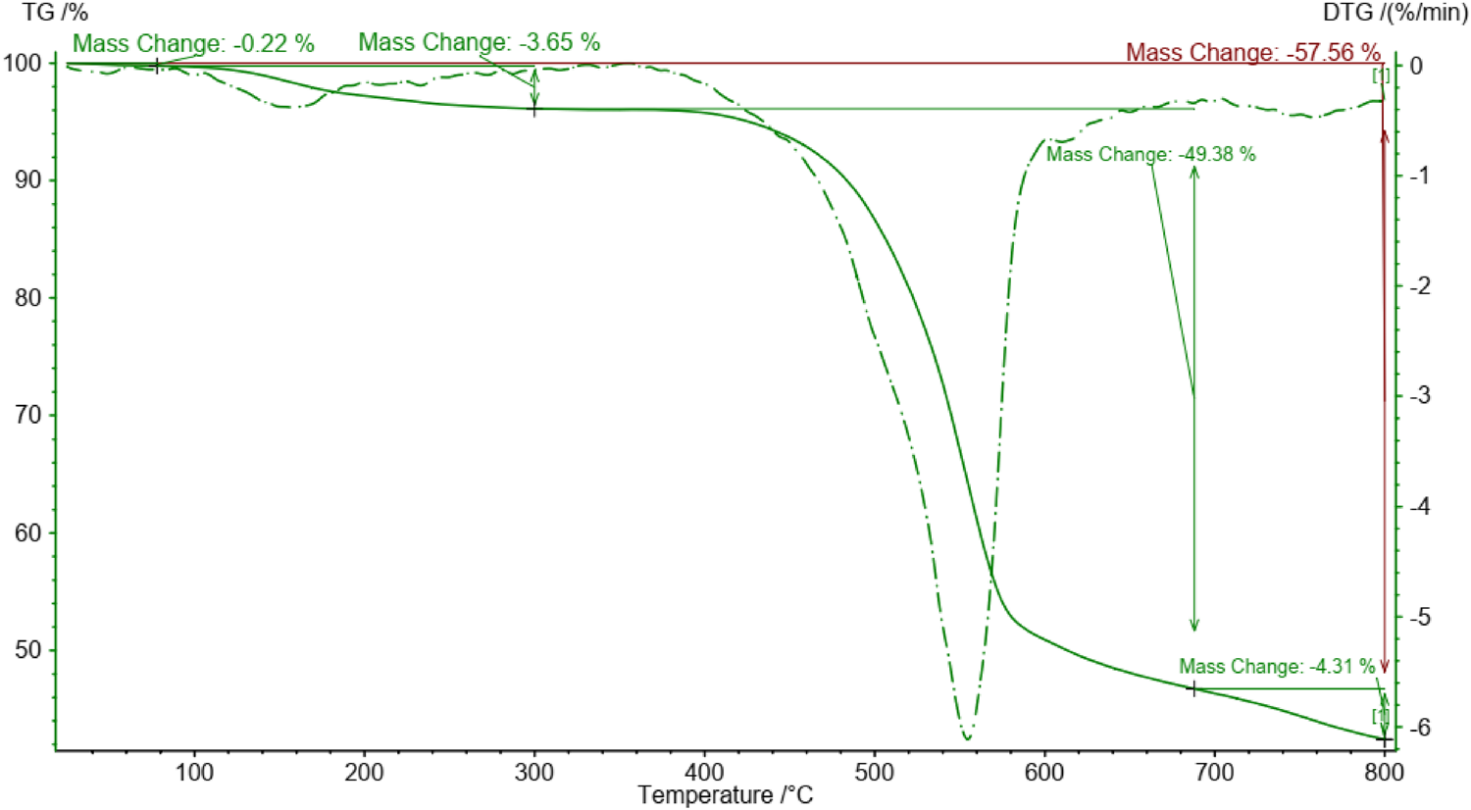
The TGA cure of FeCeO_x_@g-C_3_N_4_ nanocomposite.

Thermogravimetric analysis (TGA) is a technique used to study the thermal behavior of a material as a function of temperature. It involves measuring the weight changes of a sample as it is heated or cooled under controlled conditions (Fig. 6). The TGA of the FeCeO_x_@g-C_3_N_4_ revealed three stages of weight loss. The initial weight loss at lower temperatures due to the removal of surface-adsorbed species. These adsorbed species can include moisture, gases, or functional groups like OH groups that may be present on the composite surface. The major weight loss occurred between approximately 520–630 °C, which was attributed to the combustion of the graphitic carbon nitride phase.

To validate the applicability of ICP-MS as alternatives to EDX analysis for the measurement of Ce in the composite, FeCeO_x_@g-C_3_N_4_ nanocomposite were analyzed by ICP-MS. The amount of analyzed Ce (0.71 wt.%) corresponded to Ce content (0.69 wt.%) based on EDX measurement.

### Assessment of catalytic activity of FeCeO_x_@g-C_3_N_4_ nanocomposite for the synthesis of 2,4,5-trisubstituted and 1,2,4,5-tetrasubstituted imidazoles

After preparing and characterizing FeCeO_x_@g-C_3_N_4_, the catalytic performance of the composites was investigated for the synthesis of 1,2,4,5-tetra phenyl imidazole and 2,4,5-triphenyl imidazole. This study aimed to develop a cost-effective and easily accessible method for synthesizing these imidazole derivatives using readily available starting materials. The catalytic protocol demonstrated excellent selectivity and simplicity, allowing for the synthesis of various 2,4,5-trisubstituted and 1,2,4,5-tetrasubstituted imidazoles. The procedure presented a sustainable and chemically efficient alternative, as it utilized inexpensive and readily available starting materials.

#### Optimization of the reaction parameters for the synthesis of 1,2,4,5-tetrasubstituted imidazoles

In the optimization study, benzaldehyde, benzil, aniline, and NH_4_OAc were chosen as model substrates to prepare 1,2,4,5-tetrasubstituted imidazole. The goal was to identify the optimal reaction conditions by varying catalysts, solvents, and temperatures. Table 2 presents the results of the model reactions under different conditions. Entry 1 shows that the model reaction without a catalyst resulted in a low yield, indicating the importance of a catalyst in the reaction. Subsequently, various catalysts were tested in the model reaction (entries 2–6 and 8), and the best outcome was obtained with FeCeO_x_@g-C_3_N_4_ as the catalyst (entry 8). Further investigation was conducted to determine the optimal catalyst amount (entries 7–10). The results in Table 2 revealed that using 20 mg of FeCeO_x_@g-C_3_N_4_ as the catalyst (entry 8) provided the highest yield for synthesizing 1,2,4,5-tetraphenyl imidazoles using benzaldehyde (0.5 mmol), benzil (0.5 mmol), aniline (0.5 mmol), and ammonium acetate (0.5 mmol).

**Table 2:** The effect of various parameters on the synthesis of 1,2,4,5-tetraphenyl imidazole.

TON = 9.4 × 10^−6^ and TOF 1.3 × 10^−9^ s^−1^							
Entry	Catalysts	Catalyst (mg mol)		time (min)	Solvent	Temp. (°C)	Yield (%)^a^
1	Without catalyst	–	–	250	Ethanol	60	13
2	g-C_3_N_4_	20	0.022	150	Ethanol	60	28
3	SbCl_3_/g-C_3_N_4_	20	0.00625	150	Ethanol	60	39
4	FeCl_3_/g-C_3_N_4_	20	0.0078	150	Ethanol	60	71
5	CeCl_3_/g-C_3_N_4_	20	0.0059	150	Ethanol	60	48
6	FeCl_3_/CeCl_3_@g-C_3_N_4_	20	0.0039	150	Ethanol	60	84
7	FeCeO_x_@g-C_3_N_4_	10	0.0023	140	Ethanol	60	69
8	FeCeO_x_@g-C_3_N_4_	20	0.0047	120	Ethanol	60	98
9	FeCeO_x_@g-C_3_N_4_	35	0.007	120	Ethanol	60	98
10	FeCeO_x_@g-C_3_N_4_	50	0.0115	120	Ethanol	60	98
11	FeCeO_x_@g-C_3_N_4_	20	0.0047	120	Ethanol	25	82
12	FeCeO_x_@g-C_3_N_4_	20	0.0047	120	Water	25	78
13	FeCeO_x_@g-C_3_N_4_	20	0.0047	120	Water	60	83
14	FeCeO_x_@g-C_3_N_4_	20	0.0047	120	Methanol	25	81
15	FeCeO_x_@g-C_3_N_4_	20	0.0047	120	Methanol	60	88
16	FeCeO_x_@g-C_3_N_4_	20	0.0047	120	Acetonitrile	60	79
17	FeCeO_x_@g-C_3_N_4_	20	0.0047	120	CCl_4_	60	82
18	FeCeO_x_@g-C_3_N_4_	20	0.0047	120	Ethyl acetate	60	81
19	FeCeO_x_@g-C_3_N_4_	20	0.0047	120	DMF	60	73
20	CeO_2_@g-C_3_N_4_	20	0.0075	120	Ethanol	60	84
21	Fe_2_O_3_@g-C_3_N_4_	20	0.0079	120	Ethanol	60	48

^a^ Isolated yields.

The optimization study further investigated the effect of temperature and solvent on the synthesis of 1,2,4,5-tetrasubstituted imidazole. Table 2 provided insights into the optimal reaction conditions. Regarding temperature, the results in Table 2 (entries 8 and 11) indicated that 60 °C was the best operating temperature for the model reaction, resulting in the highest yield of the desired product (Table 2, entry 8). Moreover, the impact of different solvents on the model reaction was explored (Table 2, entries 12–19). Among the solvents tested, ethanol (EtOH) was found to be the most suitable choice for this reaction, providing favorable yields of the 1,2,4,5-tetraphenylimidazole product. Based on the results presented in Table 2, the optimized conditions for the synthesis of 1,2,4,5-tetraphenylimidazole are as follows: benzaldehyde (0.5 mmol), benzil (0.5 mmol), aniline (0.5 mmol), and ammonium acetate (0.5 mmol) as the substrates, 20 mg of FeCeOx@g-C_3_N_4_ nanocomposite as the catalyst, ethanol as the solvent, and a reaction temperature of 60 °C.

**Figure 7:**
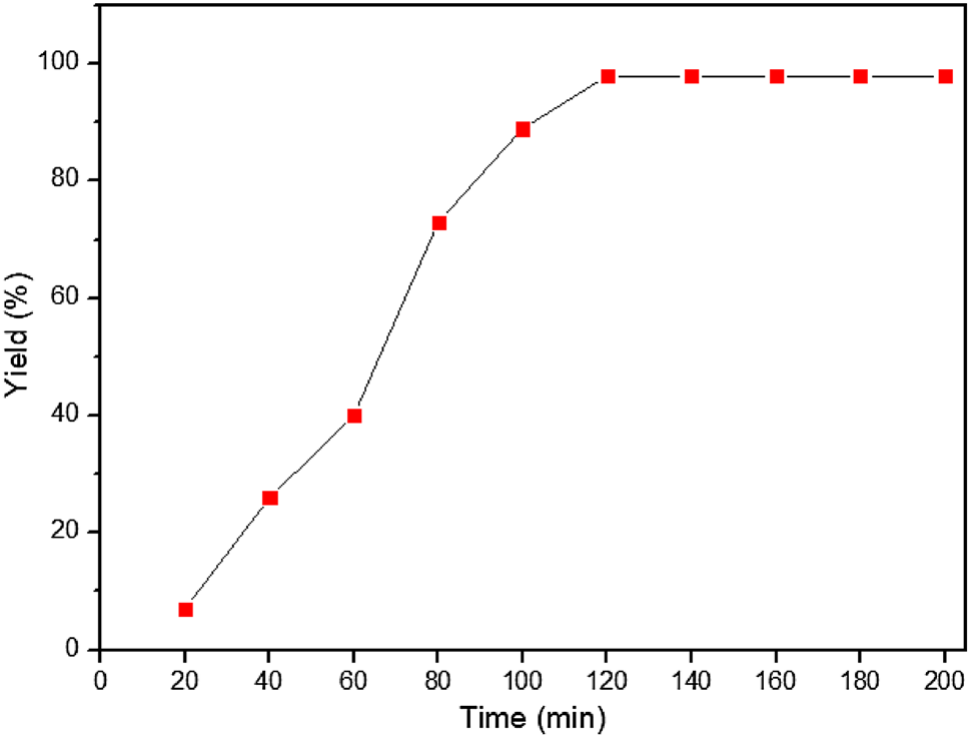
A graph of reaction time and yield on the model reaction.

The relationship between reaction time and percent yield in the model reaction was evaluated by collecting experimental data points at various reaction times. These data points were then used to plot a graph, typically referred to as Fig. 7, to visualize the trend. According to the graph, it can be observed that there is an initial increase in the percent yield as the reaction progresses. This indicates that the desired product is being formed over time, leading to an improvement in yield. However, after reaching a certain point, the graph shows a plateau, where the percent yield remains relatively constant. Beyond the plateau, a continuous increase in the yields is observed. The continuous increase in yields indicates that the reaction is proceeding in a favorable direction, leading to higher overall conversion and yield.

After optimizing the reaction conditions for synthesizing 1,2,4,5-tetraphenyl imidazole, various benzaldehydes were utilized to prepare different imidazoles. The results of these reactions are presented in Table 3, shedding light on the influence of different substituents on the benzaldehyde. The Table 3 demonstrates that both electron-withdrawing and electron-rich groups on the benzaldehyde perform well in the reaction, leading to the formation of the corresponding 1,2,4,5-tetraphenyl imidazoles in excellent yields. This suggests that a wide range of benzaldehyde derivatives can be utilized as substrates in this transformation. However, when the benzaldehyde contains an electron-donating group substituent (Table 3, entry 4), the yield of the desired product decreases. On the other hand, if the benzaldehyde possesses a strong electron-withdrawing substituent (Table 3, entry 7), the yield increases. These observations indicate that the nature of the substituents on the benzaldehyde has a significant impact on the reaction outcome. Electron-withdrawing groups tend to enhance the reactivity and favor the formation of the desired product, leading to higher yields. Conversely, electron-donating groups may hinder the reaction progress, resulting in lower yields.

**Table 3:** Synthesis of 1,2,4,5- tetra phenyl imidazole substituted in the presence of FeCeO_x_@g-C_3_N_4_ nanocomposite.


Entry	Aldehyde	Product	Time (min)	Yield (%)^a^	Melting point	
					Found	Reported
1			120	98	219–220	218–219^7^
2			130	97	160–162	159–160^2^
3			130	89	185–186	184–186^2^
4			120	92	166–168	164–166^9^
5			120	81	281–283	285–286^2^
6			120	95	156–158	154–156^10^
7			135	95	153–155	154–156^9^
8			130	94	161–163	162–164^9^
9			130	42	203–205	205–208^68^
10			130	51	244–246	247–250^57^
11			130	00	–	–
12			130	00	–	–

^a^ Isolated yields.

#### Optimization of the reaction parameters for the synthesis of 2,4,5-trisubstituted imidazoles

Based on our previous results, further optimization was carried out in the model reaction of benzaldehyde, benzil, and ammonium acetate. Reaction conditions were optimized by varying catalysts, solvents, and temperatures. The model reaction was initially carried out in the absence of the catalyst (Table 4, entry 1), leading to a low product yield. Various kinds of catalysts were used in this reaction (Table 4, entries 2–7), and the best result has been appertained to FeCeO_x_@g-C_3_N_4_. Finally, different amounts of FeCeO_x_@g-C_3_N_4_ nanocomposite were used to determine their effects on the reaction in the presence of ethanol at 80 °C (Table 4, entries 7–9). The optimum amount of catalyst was 20 mg for the synthesis of 2,4,5-triphenyl imidazole in the reaction of benzaldehyde (0.5 mmol), benzil (0.5 mmol), and ammonium acetate (1.5 mmol) (Table 4, entry 9). The model reaction was investigated at different temperatures (Table 4, entries 8–10). As a result, the best choice was 80 °C as the optimal temperature in ethanol as a solvent for this reaction. Based on Table 4 inspection, it was observed that the optimum condition for the synthesis of 2,4,5-triphenyl imidazole was benzaldehyde (0.5 mmol), benzil (0.5 mmol), ammonium acetate (1.5 mmol), and 20 mg of FeCeO_x_@g-C_3_N_4_ nanocomposite as a catalyst in ethanol as a solvent in 80 °C.

**Table 4:** The effect of various parameters on the synthesis of 2,4,5-triphenyl imidazole.

TON = 9.4 × 10^−6^ and TOF 1.3 × 10^−9^ s^−1^						
Entry	Catalysts	Amount of catalyst: mg (mol)	Time (min)	Solvent	Temp. (°C)	Yield (%)^a^
1	Without catalyst	–	220	Ethanol	80	41
2	g-C_3_N_4_	20 (0.022)	120	Ethanol	80	54
3	SbCl_3_/g-C_3_N_4_	20 (0.062)	120	Ethanol	80	61
4	CeCl_3_/g-C_3_N_4_	20 (0.078)	120	Ethanol	80	78
5	FeCl_3@_g-C_3_N_4_	20 (0.00078)	120	Ethanol	80	63
6	FeCl_3_@CeCl_3_/g-C_3_N_4_	20 (0.0059)	120	Ethanol	80	87
7	FeCeO_x_@g-C_3_N_4_	10 (0.0023)	120	Ethanol	80	92
8	FeCeO_x_@g-C_3_N_4_	20 (0.0047)	120	Ethanol	25	82
9	FeCeO_x_@g-C_3_N_4_	20 (0.0047)	120	Ethanol	60	91
10	FeCeO_x_@g-C_3_N_4_	20 (0.0047)	100	Ethanol	80	98
11	FeCeO_x_@g-C_3_N_4_	50	100	Ethanol	80	98
12	FeCeO_x_@g-C_3_N_4_	20 (0.0047)	120	Water	25	64
13	FeCeO_x_@g-C_3_N_4_	20 (0.0047)	120	Water	80	81
14	FeCeO_x_@g-C_3_N_4_	20 (0.0047)	120	Methanol	25	78
15	FeCeO_x_@g-C_3_N_4_	20 (0.0047)	120	Methanol	80	90
16	FeCeO_x_@g-C_3_N_4_	20 (0.0047)	120	Acetonitrile	25	68
17	FeCeO_x_@g-C_3_N_4_	20 (0.0047)	120	Acetonitrile	80	82
18	FeCeO_x_@g-C_3_N_4_	20 (0.0047)	120	Ethyl acetate	25	66
19	FeCeO_x_@g-C_3_N_4_	20 (0.0047)	120	Ethyl acetate	80	84

^a^ Isolated yields.

After determining the optimal conditions in the model reaction, various aldehydes, including aromatic, heteroaromatic, and aliphatic types, were employed to synthesize different imidazoles under the optimized conditions. The results of these reactions are presented in Table 5. In general, benzaldehydes with various substituents, whether electron-withdrawing or electron-donating groups, exhibited good reactivity and provided the corresponding 2,4,5-trisubstituted imidazoles in moderate to good yields. This suggests that a wide range of benzaldehydes can be utilized as substrates in this reaction, allowing for the incorporation of diverse substituents into the imidazole framework. The yields of the reactions were further influenced by the nature of the substituents on the benzaldehyde. When benzaldehydes with strong electron-withdrawing substituents like NO_2_ were used (Table 5, entries 7 and 9), the reaction yields were increased. This indicates that electron-withdrawing groups enhance the reactivity and favor the formation of the desired products, leading to higher yields. However, when aliphatic aldehydes were employed (Table 5, entry 11), the yields of the corresponding imidazoles were low. This suggests that aliphatic aldehydes may not be as suitable for this transformation under the given optimized conditions.

**Table 5:** Synthesis of 2,4,5- triphenyl imidazole substituted in the presence FeCeO_x_@g-C_3_N_4_ nanocomposite.


Entry	Aldehyde	Product	Time (min.)	Yield (%)^a^	Melting point	
					Found	Reported
1			80	98, (97)^b^	276–278	276–278^2^
2			80	95	228–230	230–231^7^
3			80	96	272–274	274–276^2^
4			100	78	264–266	266–268^2^
5			90	94	262–263	262–264^7^
6			90	91	188–189	190–192^7^
7			100	81	239–240	240–242^2^
8			90	93	256–258	258–260^7^
9			100	72	229–230	228–230^11^
10			90	92	261–263	262–264^48^
11			100	78	228–230	225–230^57^
11			100	29	168–170	169–171^14^

^a^ isolated yields. Yields for five gram scale.

### Recycle experiments

**Figure 8:**
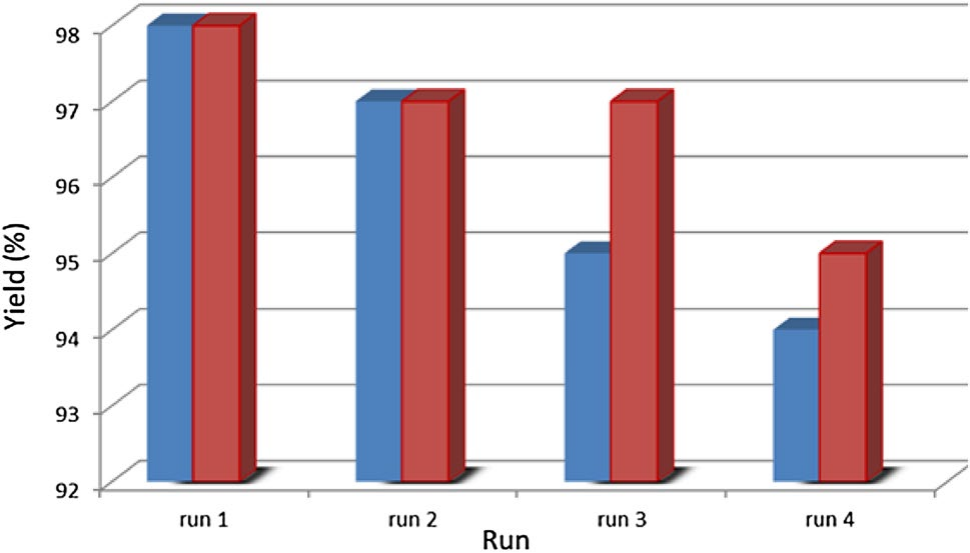
Recyclability of FeCeO_x_@g-C_3_N_4_ nanocatalyst in the preparation of tri substituted imidazole (red chart) and four substituted imidazole (blue chart).

Recyclability is an important aspect of catalysts in terms of green chemistry, and experiments were conducted on a larger scale (2.5 mol) to reduce system errors and evaluate the catalyst's reusability. In this case, 100 mg of the FeCeO_x_@g-C_3_N_4_ nanocomposite catalyst was used for 5 mol of starting materials. After the completion of the reaction, 10 mL of ethyl acetate was added to the reaction mixture, and the catalyst was separated from the mixture using centrifugation. The separated catalyst was then washed with ethyl acetate. The washed catalyst was successfully reused for four consecutive runs of reactions without any significant decrease in reaction yields. As shown in Fig. 8, the yields of the four runs for the synthesis of 2,4,5-trisubstituted imidazoles (red column) were 98%, 97%, 97%, and 95%. Similarly, the yields for the four runs of synthesis of 1,2,4,5-tetrasubstituted imidazoles (blue column) were 98%, 97%, 95%, and 94%, respectively. The amount of catalyst remaining after the five runs was 93 mg, indicating a minor loss of catalyst during the recycling process.

To further confirm the stability of the FeCeO_x_@g-C_3_N_4_ nanocomposite, TGA analysis and FTIR spectroscopy were performed on both the fresh and recycled catalyst. The results from TGA analysis and FTIR spectra showed no appreciable changes in the chemical structure of the recycled FeCeO_x_@g-C_3_N_4_ after five cycles, further indicating its stability and suitability for reuse (Supporting information).

Table 6 provides a comparison of the catalytic efficiency of different methods reported in the literature for the synthesis of tetrasubstituted imidazoles. The FeCeO_x_@g-C_3_N_4_ catalyst is specifically evaluated in the model reaction and compared to other catalysts used in similar reactions. The FeCeO_x_@g-C_3_N_4_ catalyst demonstrated excellent activity and outperformed the other catalysts in the set (58–60,66,68).

**Table 6:** Comparison of the catalytic efficiency of various catalyst in the literature.

Entry	Catalyst	Solvent	Time (h)	Yield (%)	Temp. (°C)	Reusability	References
1	B(OH3)	MeOH	8	60–97	60	No	^61^
2	HPVAC-20	IL	1	86–94	120	Yes	^1^
3	SLS	Water	2	80–90	80	No	^62^
4	Dendrimer-PWA^n^	–	1	82–96	90	Yes	^63^
5	MCS-GT@Co(II)	EtOH	5	67–99	Reflux	Yes	^13^
6	H-BEA(15)	–	1	81–99	100	Yes	^7^
7	[bmim]_3_[GdCl_6_]	IL	2.5	88–95	120	Yes	^64^
8	DBSA	Water	4	73–86	Reflux	No	^65^
9	Cu@imine/Fe_3_O_4_	–	0.5	93–98	80	Yes	^67^
10	FeCeOx@g-C_3_N_4_	Ethanol	2	81–98	60	Yes	This work

**Figure 9:**
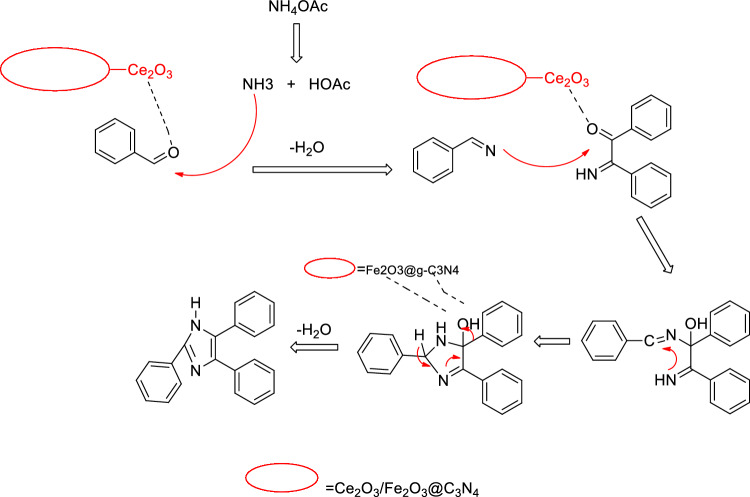
Proposed mechanism of the synthesis of trisubstituted imidazoles.

A reasonable mechanism for the synthesis of trisubstituted imidazoles using the FeCeO_x_@g-C_3_N_4_ nanocomposite as a catalyst is illustrated in Fig. 9.

The mechanism can be described as follows: The reaction begins with the condensation of an aldehyde and ammonium acetate in the presence of FeCeO_x_@g-C_3_N_4_ catalyst. The catalyst facilitates the formation of an imine intermediate through the nucleophilic addition of the amine group of ammonium acetate to the carbonyl group of the aldehyde. The imine intermediate then undergoes a subsequent reaction with a benzyl compound. This reaction can involve the nucleophilic attack of the nitrogen atom in the imine intermediate on the electrophilic carbon atom of the benzyl compound. Following the nucleophilic attack, a rearrangement occurs, leading to the formation of a trisubstituted imidazole. This rearrangement step involves the migration of substituents within the intermediate, resulting in the desired trisubstituted imidazole product. It is important to note that cerium oxide (CeO_2_) plays a crucial role in this transformation. The presence of cerium oxide in the FeCeO_x_@g-C_3_N_4_ nanocomposite likely enhances the catalytic activity and stability of the catalyst. Additionally, the FeCeO_x_@g-C_3_N_4_ nanocomposite exhibits a synergistic effect, leading to increased yields of the trisubstituted imidazole product (Fig. 9).

## Conclusion

In summary, the FeCeO_x_@g-C_3_N_4_ nanocomposite is synthesized by calcinating melamine and immobilizing Ce(III) and Fe(III) on graphitic carbon nitride (g-C_3_N_4_). This iron-based nanocomposite, in combination with cerium functionality and the good surface area of g-C_3_N_4_, shows great potential for one-pot preparations of imidazole derivatives, resulting in good to excellent yields within short reaction times. The heterogeneous catalyst is easily separated and can be reused in subsequent reactions. This nanocomposite offers several advantages as a catalyst for imidazole synthesis, including its efficient performance, easy separation, and recyclability.

## Experimental

### Materials and chemicals

Melting points were measured in open capillaries with the Buchi 535 melting-point apparatus. The reactions were monitored by thin-layer chromatography (TLC) with UV light as detecting agents. EDS spectra and Scanning Electron Microscope (SEM) images were prepared via the TESCAN Vega3 Model. Powder X-ray diffraction (XRD) analyses were given in a Bruker AXS-D_8_ Advance diffractometer. Fourier transfer infrared spectroscopy (FT-IR) in Shimadzu IR-460. ^1^H NMR spectra were recorded on a 500 MHz spectrometer and ^13^C NMR spectra on a 125 MHz NMR spectrometer, respectively, using CDCl3 or DMSO(D6) as a solvent; chemical shifts have been expressed in ppm downfield from TMS.

### Catalyst preparation

#### Synthesis of g-C_3_N_4_

According to our previous paper, the bulk g-C_3_N_4_ was synthesized by directly heating melamine in air methods^43,44^. 10 g of melamine powder was placed in a covered 50 mL alumina crucible and then heated in a muffle furnace at a ramp rate of 5 °C/min and kept for three h at 550 °C in air. After cooling to room temperature, a light yellow powder was collected and stored for further use. The g-C_3_N_4_ nanosheets are prepared by thermal exfoliation in the air. In detail, 2 g of bulk g-C_3_N_4_ was put into an uncovered crucible for heat treatment at 550 °C for 3 h to obtain white powder.

#### Synthesis of FeCeO_x_@g-C_3_N_4_ nanocomposite

At first, the g-C_3_N_4_ (400 mg), which was synthesized in the previous step, was placed in a 200 mL Erlenmeyer and then dispersed in 100 mL of methanol/water (1:1) using sonication for 10 min at room temperature. Then, 40 mg CeCl_3_ and 40 mg FeCl_3_ was dispersed in methanol, and 20 mL NaOH (2 M) was added to the mixture and sonicated for 10 min. Henceforward, the mixture was stirred for 5 h at 60 °C, and the mixture was filtered and washed with 10 mL of methanol and dried overnight at 60 °C under vacuum to obtain FeCeO_x_@g-C_3_N_4._

### General procedure for synthesizing 1,2,4,5-tetraphenyl imidazole

To a mixture of aniline (0.5 mmol), benzaldehyde (0.5 mmol), benzil (0.5 mmol) and ammonium acetate (0.5 mmol), FeCeO_x_@g-C_3_N_4_ (20 mg) as catalyst and ethanol (1 mL) as a solvent were added in a 5 mL round-bottomed flask respectively. The reaction mixture was stirred with a stirrer at 60 °C for 2 h, and TLC monitored the progress of the reaction. After the reaction was completed, 20 mL of ethyl acetate was added, the catalyst was removed by centrifuge, and the catalyst was washed with ethyl acetate and reused for the subsequent reactions. The organic residue was recrystallized to obtain 1,2,4,5-tetraphenyl imidazole and derivatives as pure products.

### General procedure for the synthesis of 2,4,5-triphenyl imidazole

A mixture of benzaldehyde (0.5 mmol), benzil (0.5 mmol), ammonium acetate (1.5 mmol), FeCeO_x_@g-C_3_N_4_ (20 mg) as a catalyst, and ethanol (1 mL) as a solvent were placed in a 5 mL round-bottomed flask, respectively. The reaction mixture was stirred with a stirrer at 80 °C for 100 min, and TLC monitored the progress of the reaction. After the reaction was completed, 10 mL of ethyl acetate was added, and the catalyst was removed by centrifuge. Then, the catalyst was washed with hot ethanol and ethyl acetate and reused for the subsequent reactions. The ethanolic residue was recrystallized to obtain 2,4,5-triphenyl imidazole and derivatives as pure products.

### Supplementary Information

Supplementary Figures.

## Data Availability

The data that support the findings of this study are available on request from the corresponding author.
