# Supplementary material for: Facile synthesis of FeCeOx nanoparticles encapsulated carbon nitride catalyst for highly efficient and recyclable synthesis of substituted imidazoles
Source: Sci Rep. 2023 Oct 14;13:17474. doi: 10.1038/s41598-023-44747-7 (PMC10576832; doi:10.1038/s41598-023-44747-7)

**Supporting information**

**Facile Synthesis of FeCeO_x_ Nanoparticles Encapsulated Carbon Nitride Catalyst for Highly Efficient and Recyclable Synthesis of Substituted Imidazoles**

Najmedin Azizi,^*^ Mostafa Saadat, Mahtab Edrisi

^1^Chemistry & Chemical Engineering Research Center of Iran, P.O. Box 14335-186, Tehran, Iran

Email: azizi@ccerci.ac.ir


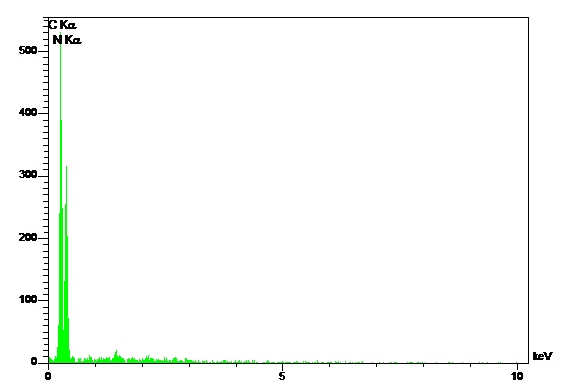


The EDS analysis of g-C3N4 nanocomposite


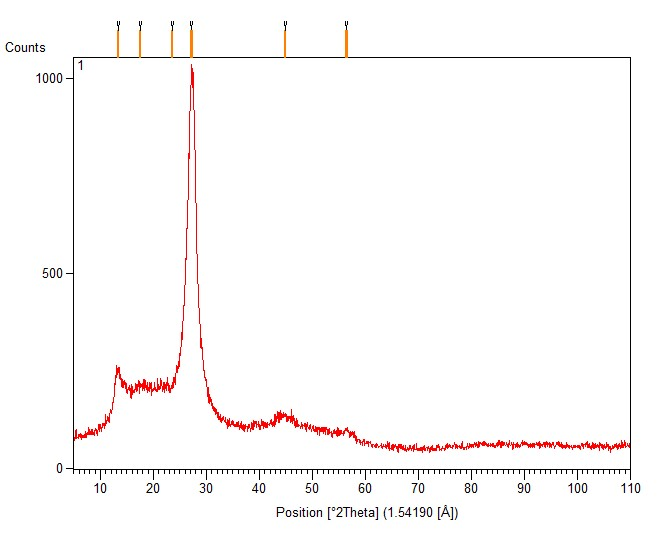


The XRD analysis of pure g-C3N4


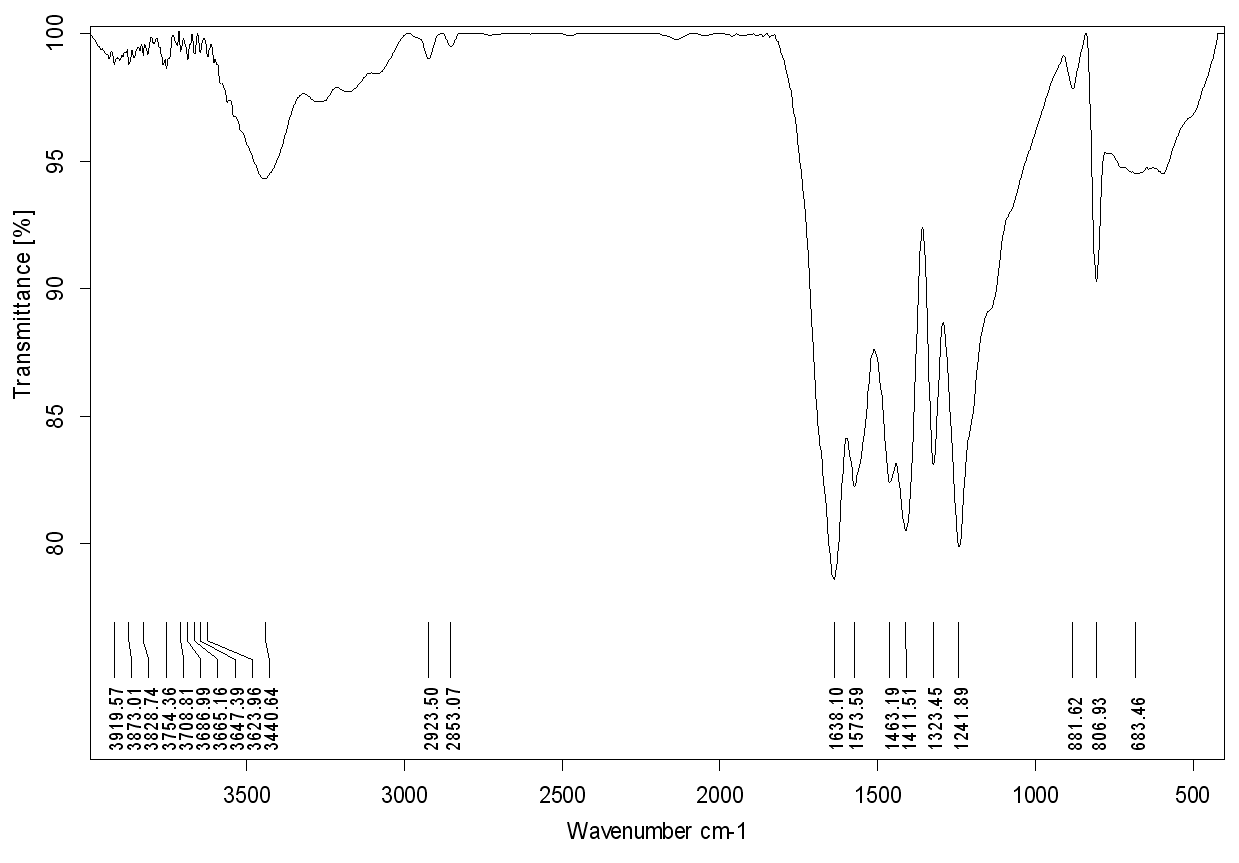


The FT-IR spectra of pure g-C3N4


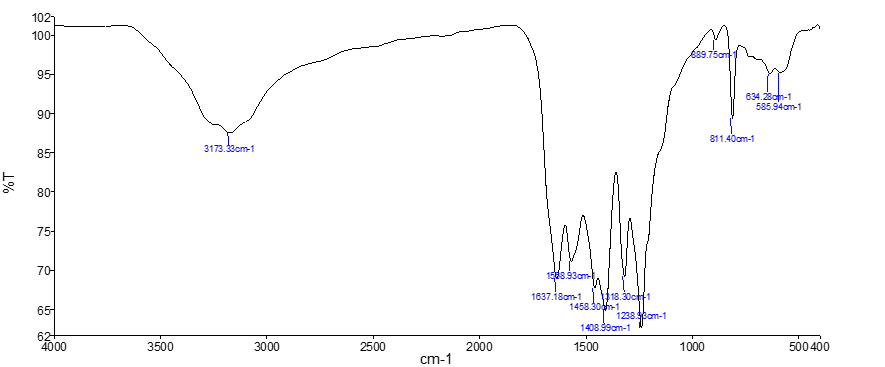


The FT-IR spectra of reused FeCeOx@g-C3N4 nanocomposite


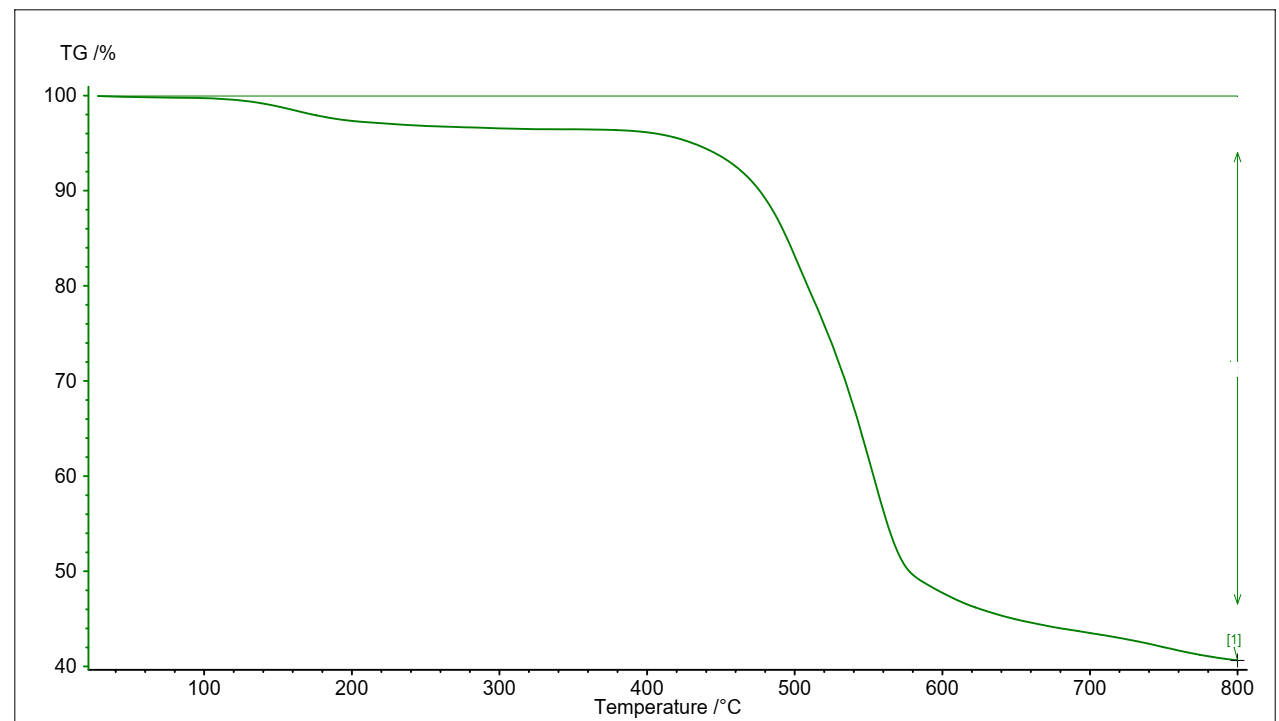


The TGA cure of reused FeCeO_x_@g-C_3_N_4_ nanocomposite


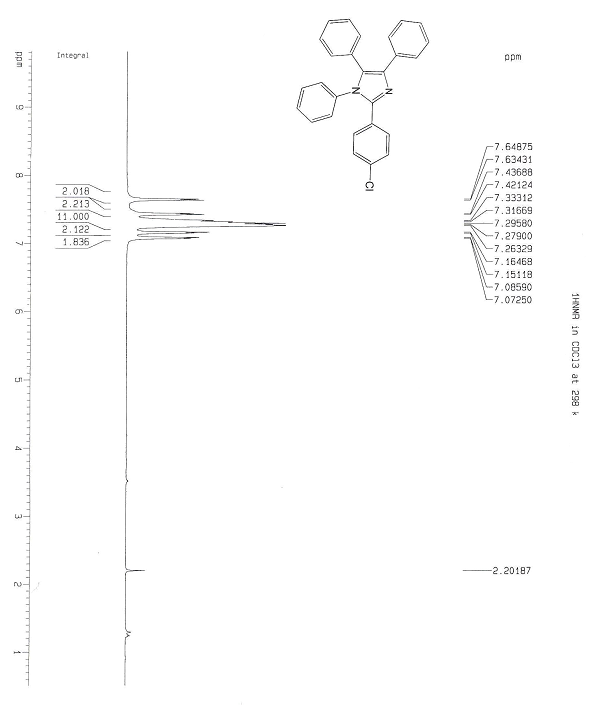


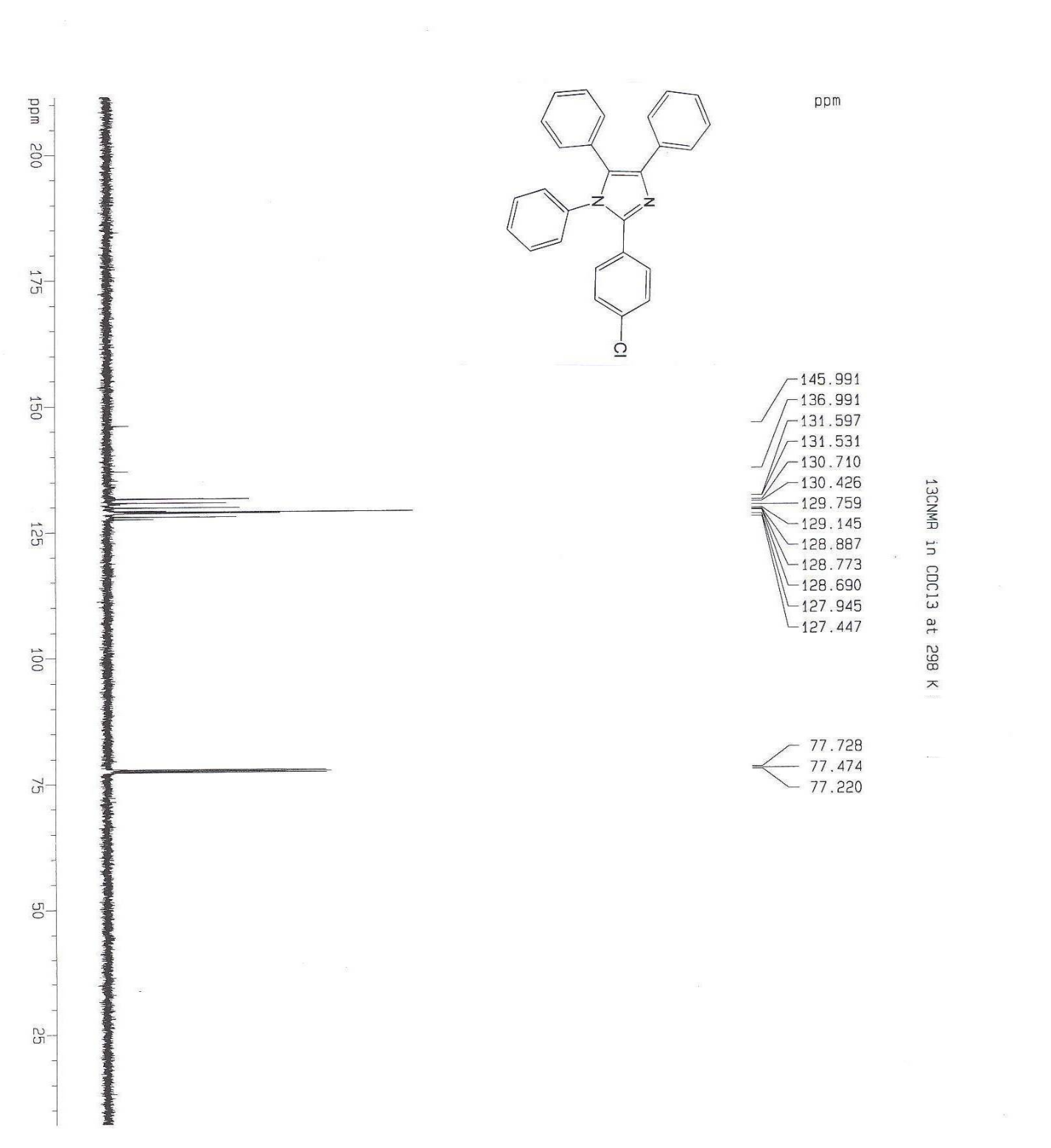


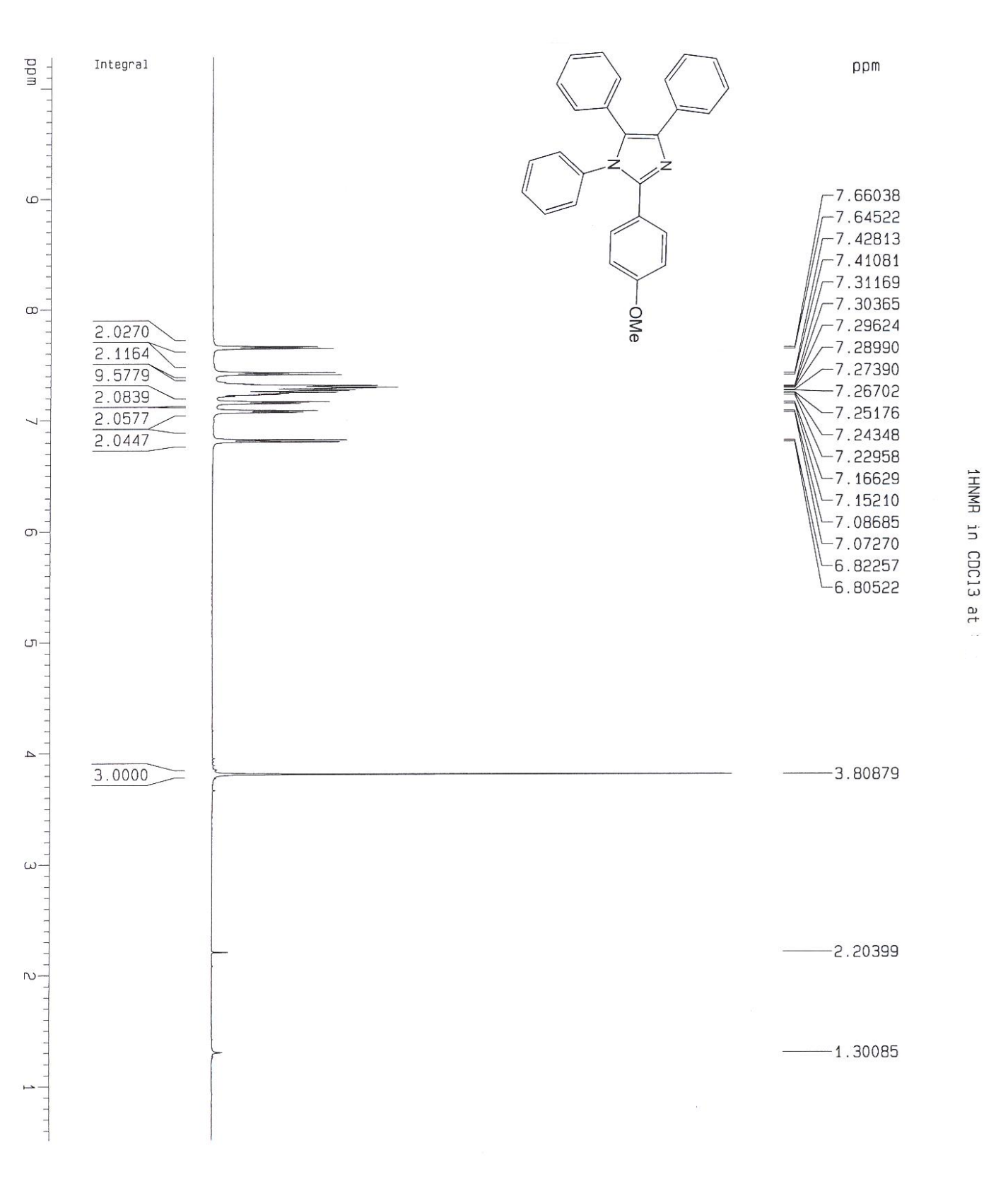


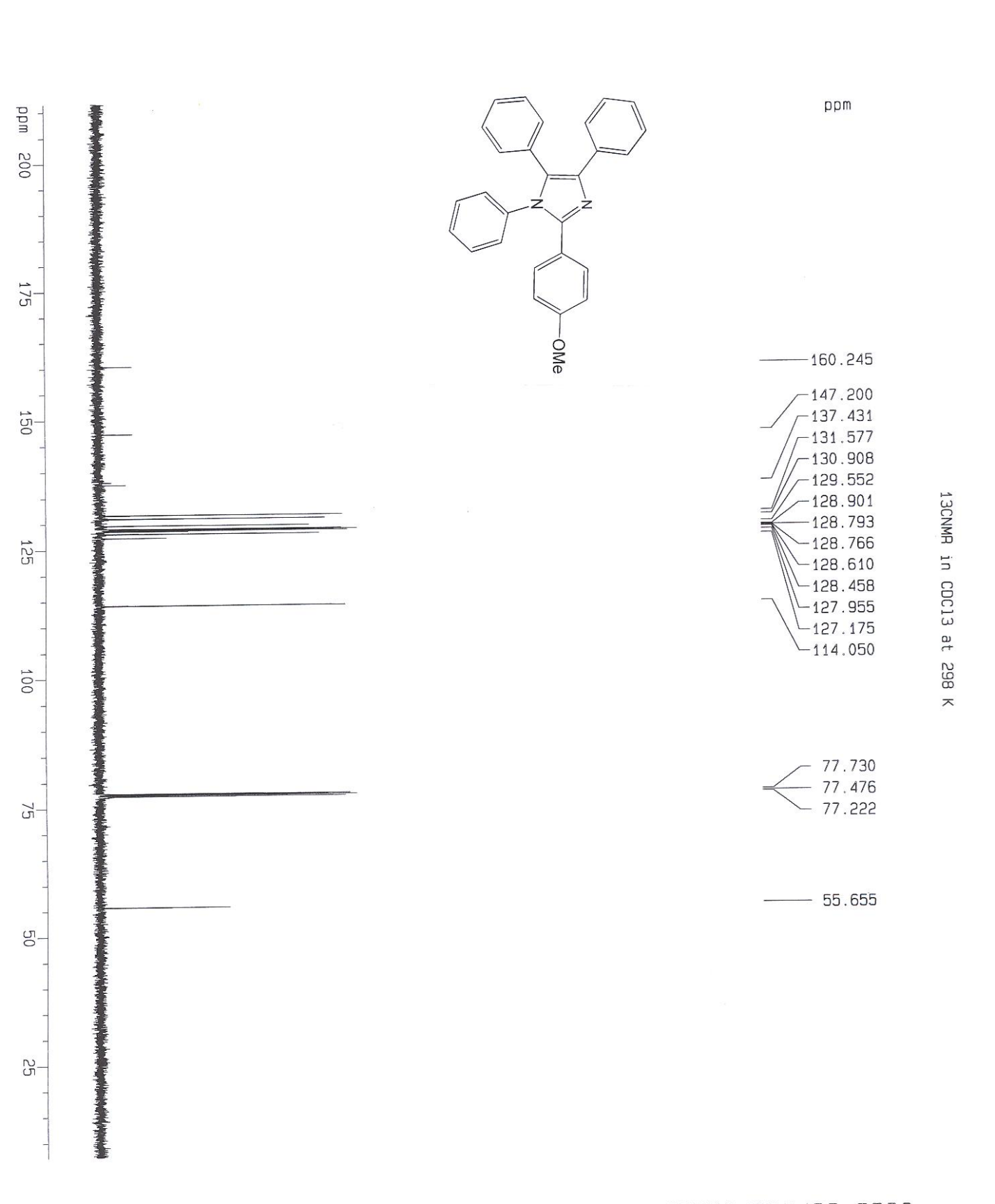


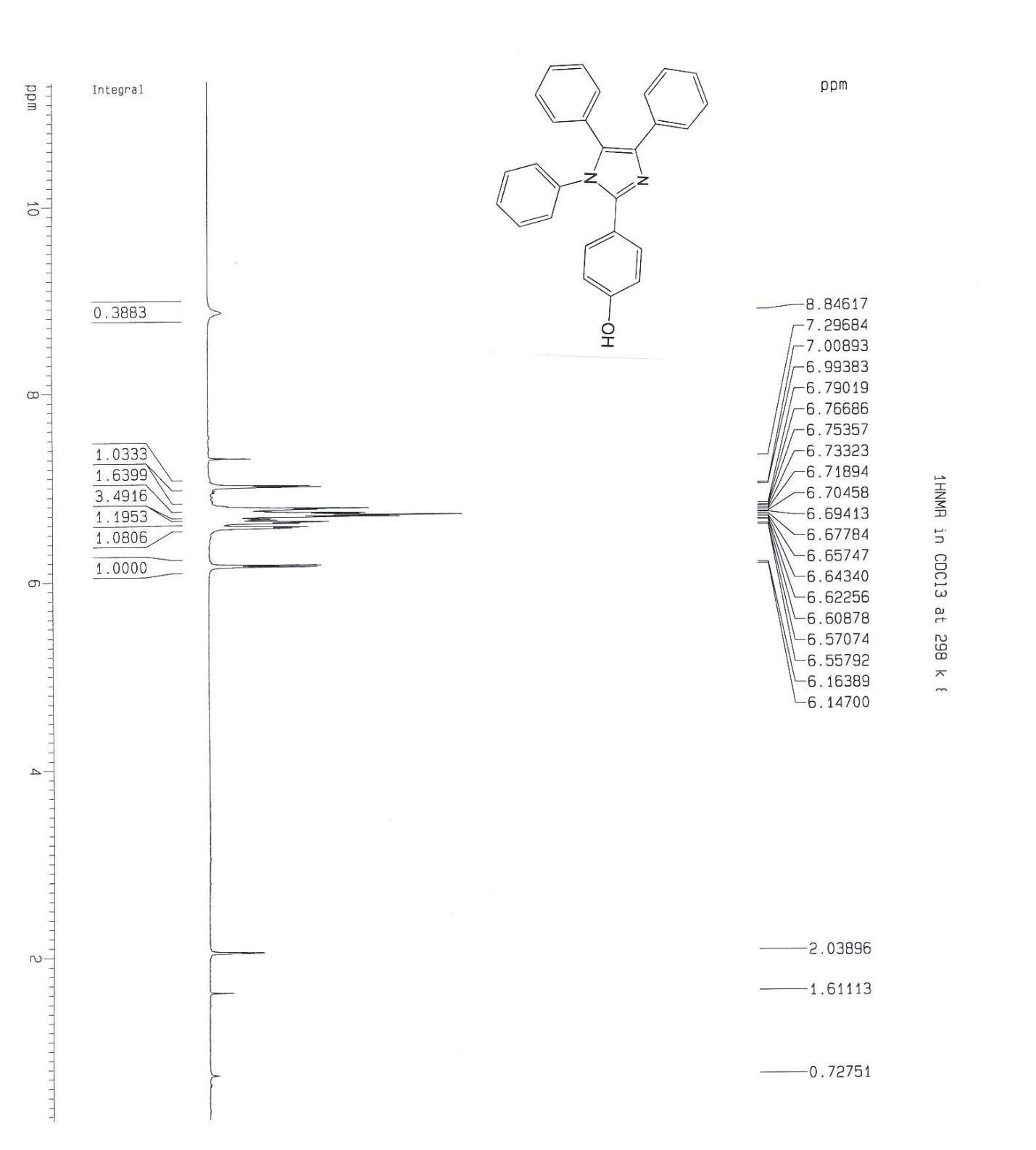


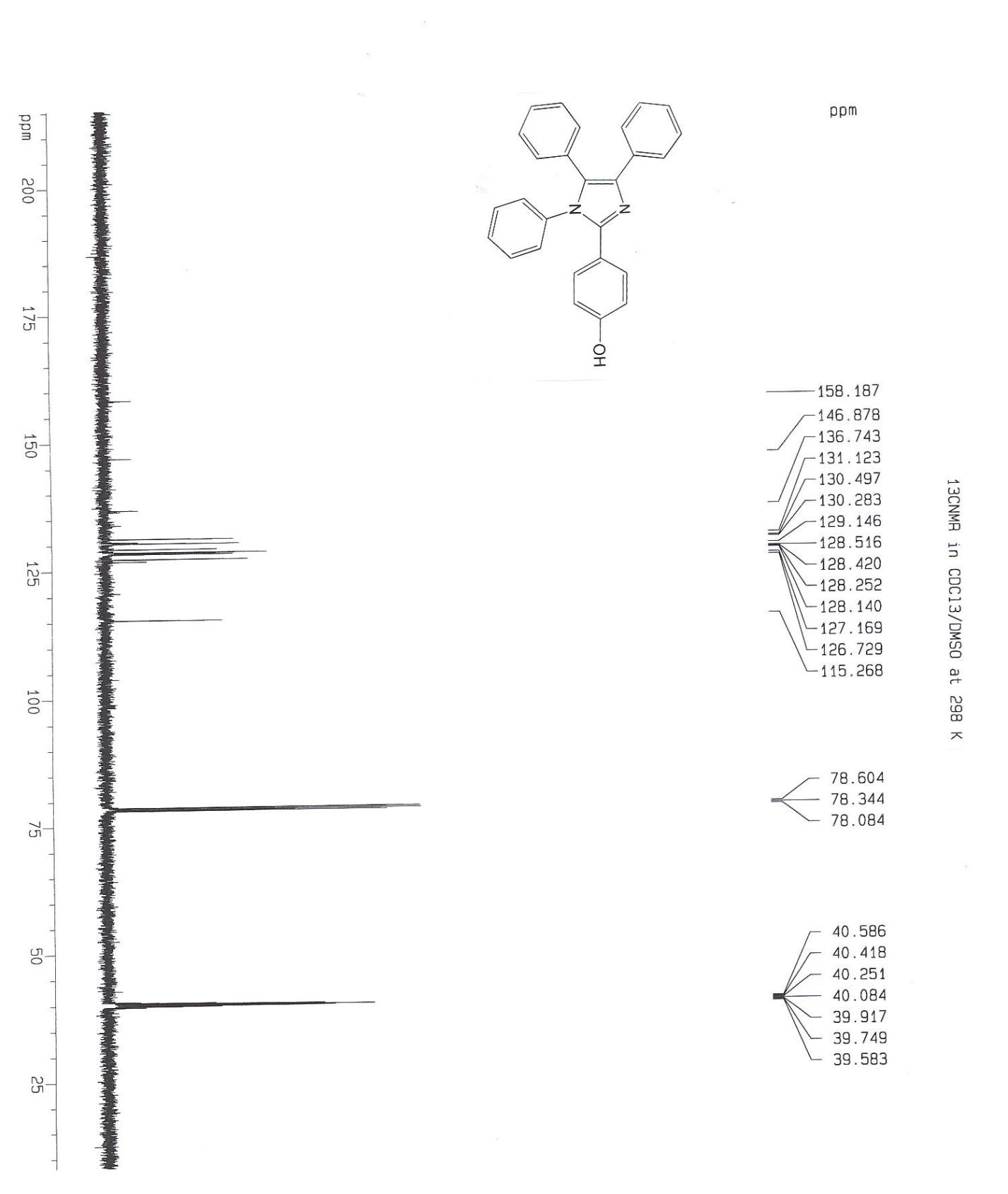


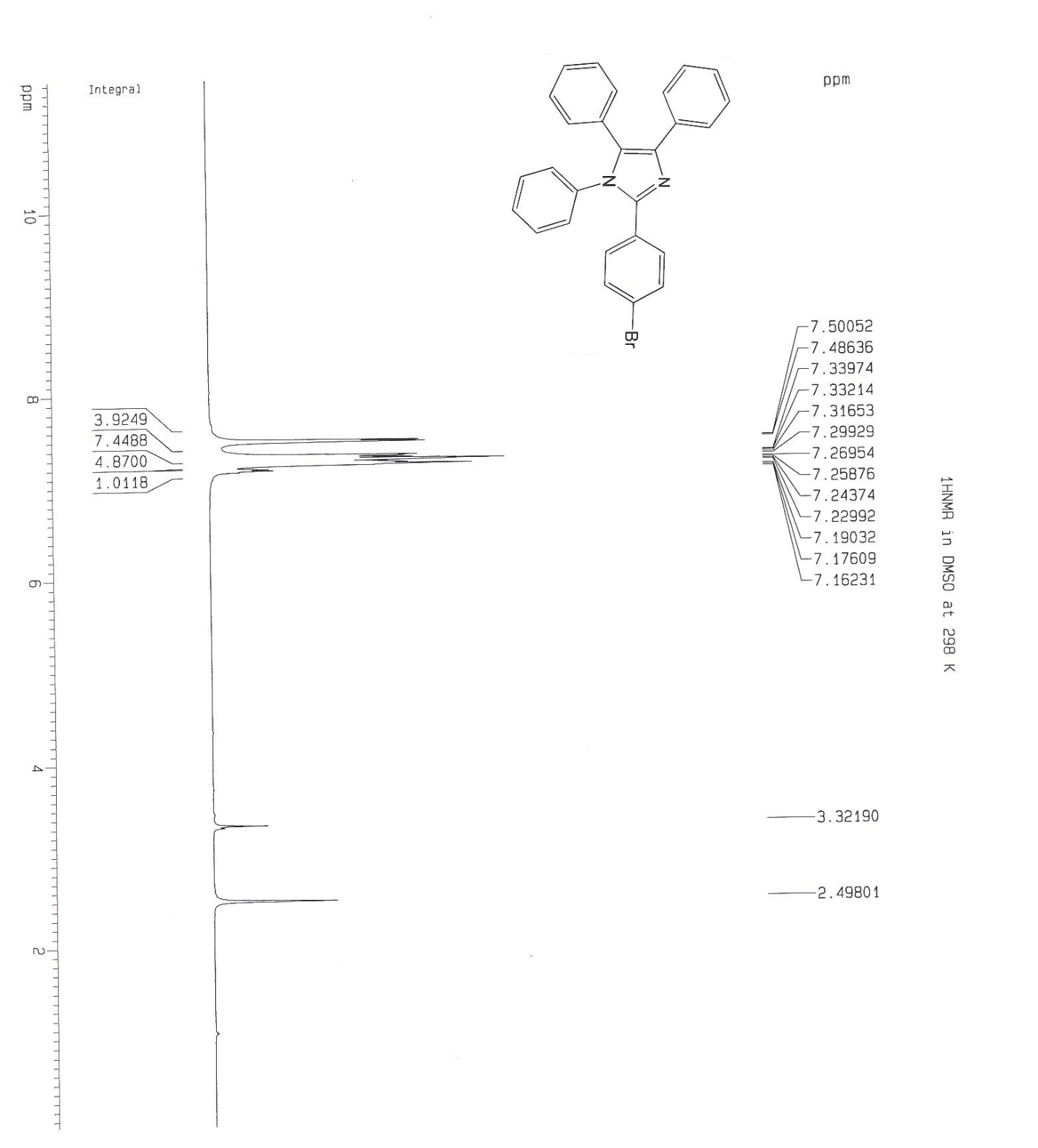


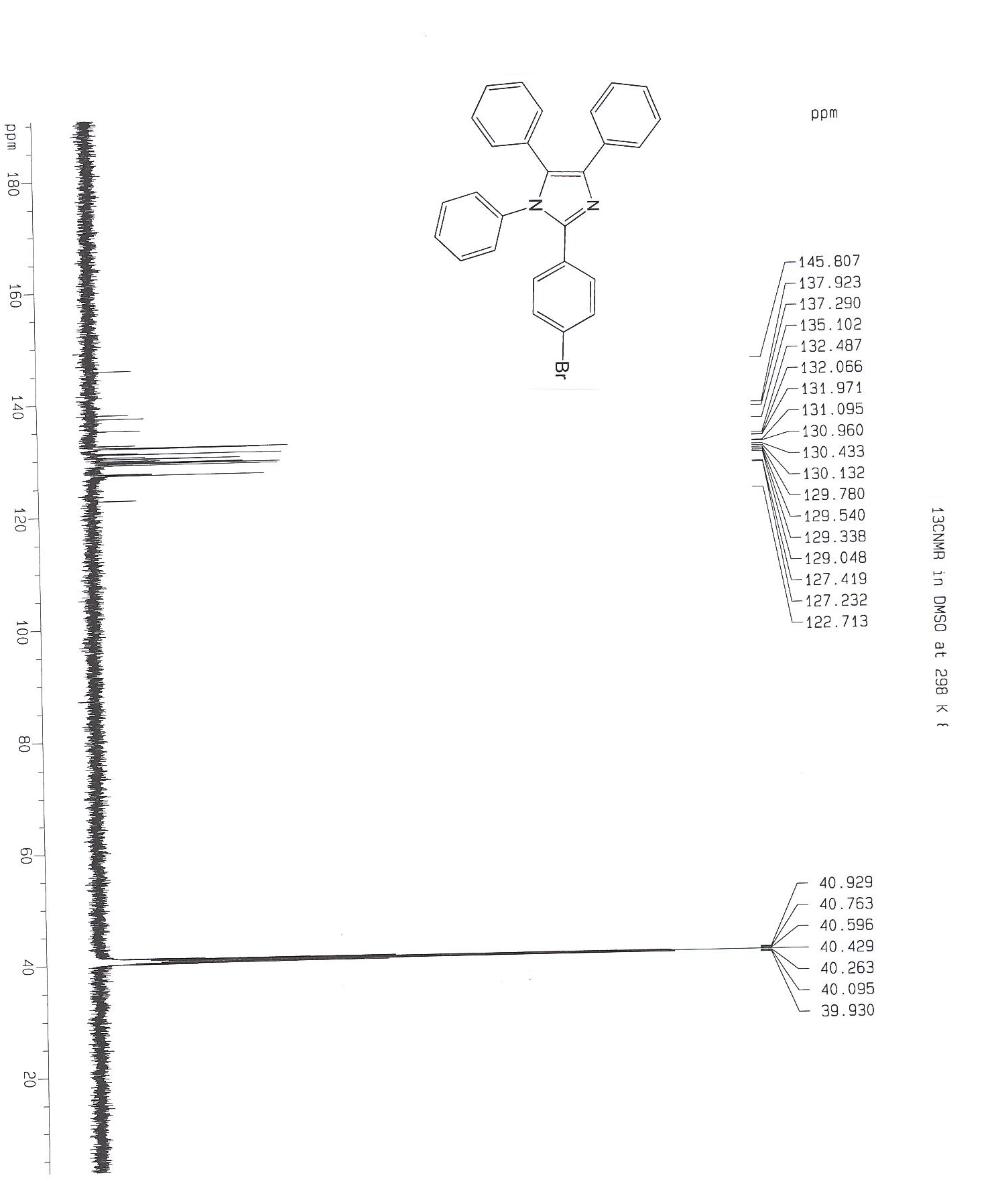


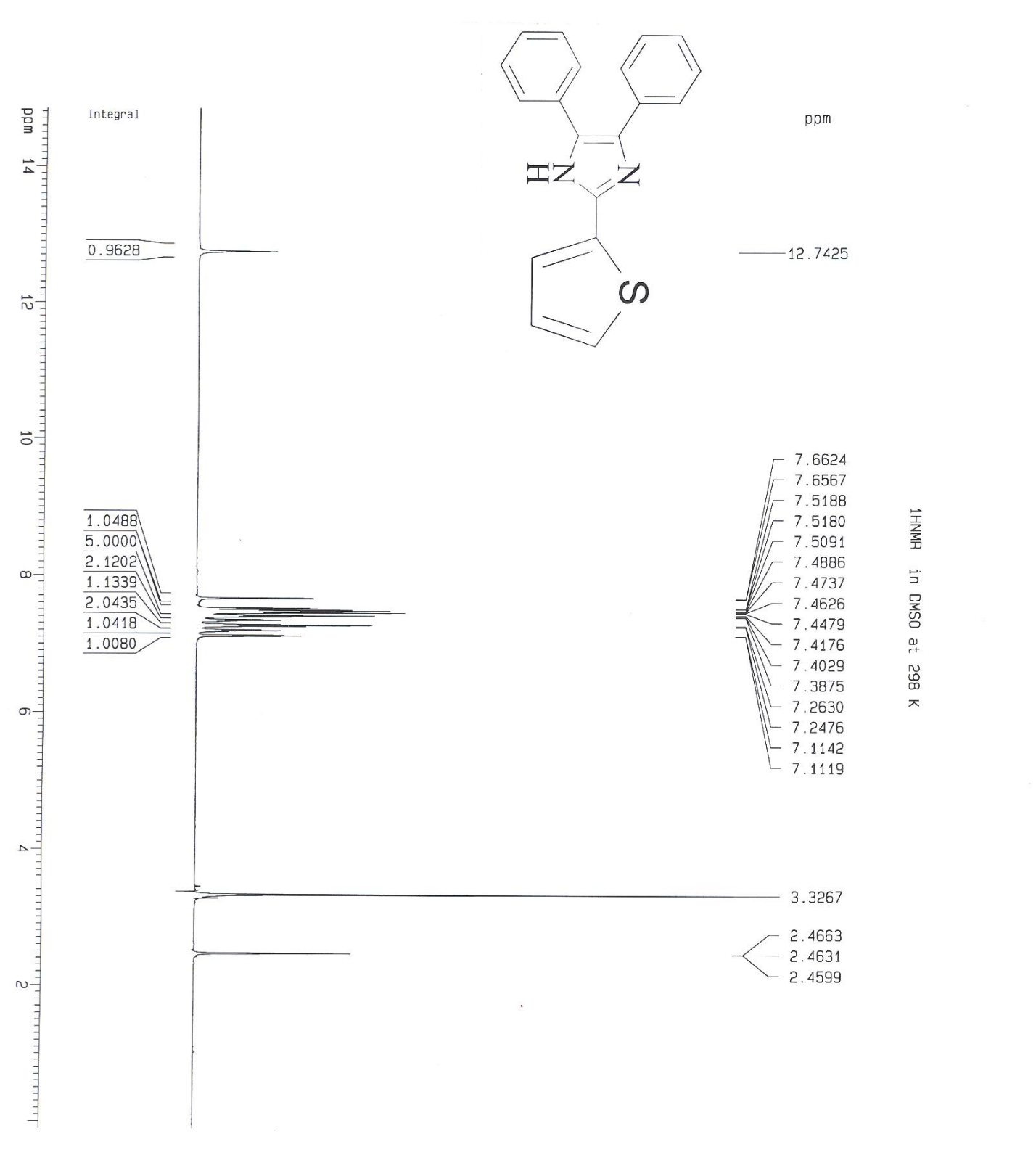


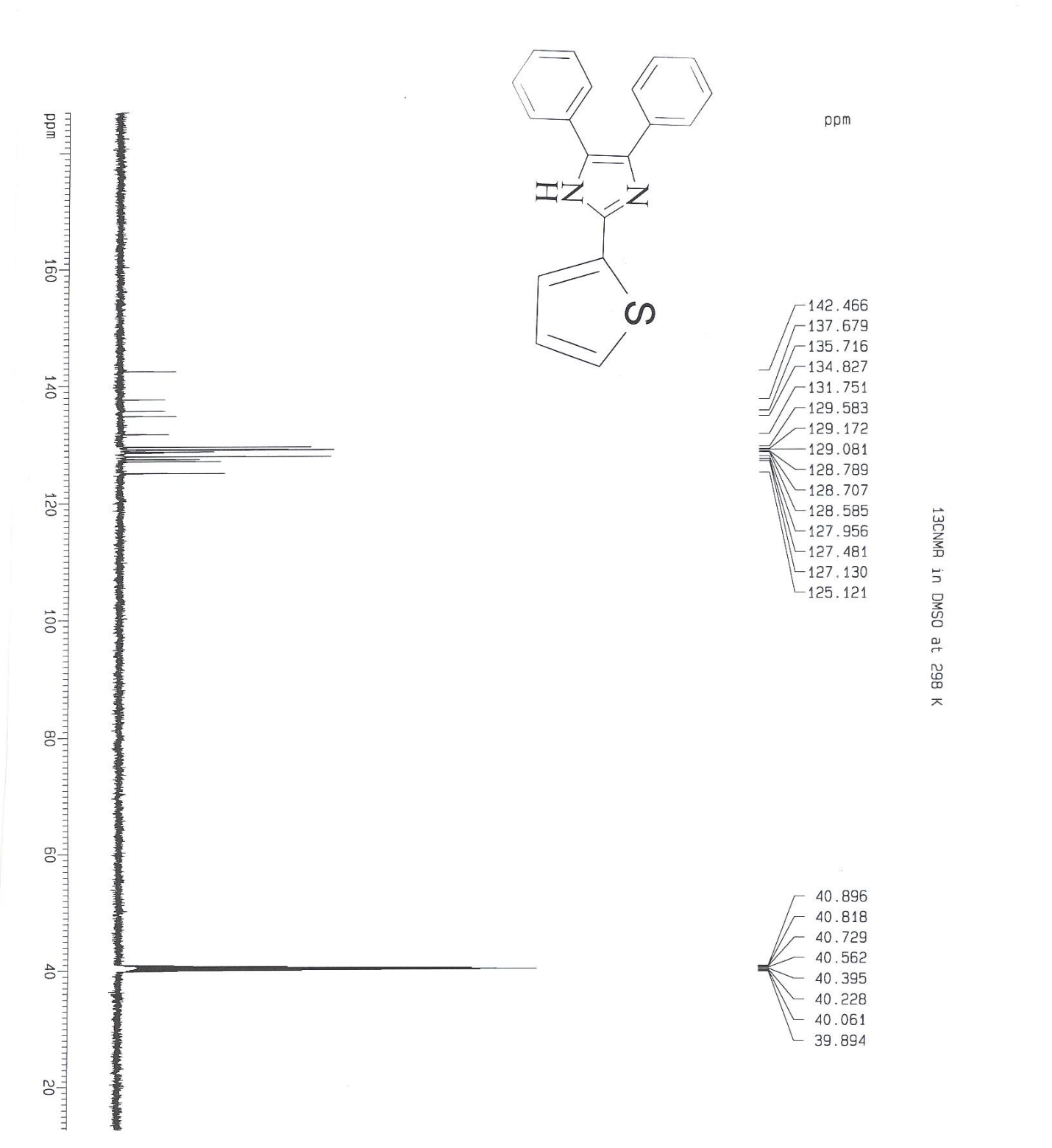

Supplement: Supplementary file 1 — Supplementary Figures. [file 41598_2023_44747_MOESM1_ESM.docx]
